# Graphene nanosheets derived from plastic waste for the application of DSSCs and supercapacitors

**DOI:** 10.1038/s41598-021-83483-8

**Published:** 2021-02-16

**Authors:** Sandeep Pandey, Manoj Karakoti, Karan Surana, Pawan Singh Dhapola, Boddepalli SanthiBhushan, Swaroop Ganguly, Pramod K. Singh, Ali Abbas, Anurag Srivastava, Nanda Gopal Sahoo

**Affiliations:** 1 Department of Chemistry, Professor Rajendra Singh Nanosciene and Nanotechnology Centre, DSB Campus, Kumaun University, Nainital, Uttarakhand 263001 India; 2 Center of Excellence on Solar Cells & Renewable Energy, Department of Physics, School of Basic Sciences and Research, Sharda University, Greater Noida, Uttar Pradesh 201308 India; 3 Department of Electrical Engineering, Indian Institute of Technology Bombay, Mumbai, 400076 India; 4 School of Chemical and Biomolecular Engineering, The University of Sydney, Sydney, NSW Australia; 5 Atal Bihari Vajpayee Indian Institute of Information Technology and Management, Gwalior, Madhya Pradesh 474015 India

**Keywords:** Energy science and technology, Materials science, Nanoscience and technology

## Abstract

The present study reports the upcycling process of waste plastics into value-added product graphene nanosheets (GNs) and their subsequent applications in dye sensitized solar cells (DSSCs) and supercapacitors. Bentonite nanoclay has been used as an agent for the degradation of waste plastics with two step pyrolysis processes at 450 °C and 945 °C in an inert atmosphere of N_2_ gas to obtain GNs. The GNs with few layers were confirmed by the RAMAN spectroscopy, XRD and HRTEM analyses. Further, FT-IR and EDX analyses also performed for the identification and quantitative analysis of functional groups in GNs. The GNs thus synthesized from plastic waste have been used for the fabrication of DSSCs and supercapacitors. The DSSC fabrication with GNs as part of photo-anode with polymeric electrolyte showed a high fill factor of 86.4% and high V_oc_ of 0.77 V, which were also supported by the computational findings. On the other hand, the utilization of GNs as an active layer material of supercapacitor electrodes offered a high specific capacitance of 398 F/g with a scan rate of 0.005 V/s. The supercapacitor also exhibited significant energy density (E_d_) and power density (P_d_) of 38 Wh/kg and 1009.74 W/kg, respectively. Thus, the process illustrated the utility of waste plastics upcycling for conservation of EEE i.e., ecology, economy and energy for better tomorrow.

## Introduction

The demand for sustainable green energy and eco-friendly upcycling of plastic waste with attractive industrial symbiosis and cost benefit analysis are the biggest challenges of the twenty first century. Both these issues must be resolved in order to address the problems associated with the production of clean energy and sustainable protection of the ecology and economy from plastic waste. In a circular economy, transition is a common factor, and the word “waste” can be replaced by “resource material” as the waste is used as feedstock materials in various processes^1^. In this way, the waste materials act as resource materials, and cascade proper synergistic economic and societal benefits along with sustainable environmental management^2–7^. The future of sustainable environmental management also depends on the scale of deployment of the renewable energy production and storage technology. In this regard, the production of solar energy through utilization of photovoltaic technology is one of the most prominent ways to generate sustainable green energy^8^.

Among photovoltaic technologies, the dye sensitized solar cell (DSSC) stands as a promising candidate for the production of greener energy due to its ease of fabrication^9^. Yet, the research on DSSC is mainly focused for the outdoor applications. The development of DSSC for indoor applications through materials that can harvest energy even in the presence of dim lighting is an important research aspect^10^. Hence, the research for developing DSSCs using greener and cost-effective materials is on the rise. In the last decade, the incorporation of conducting polymers, transition metal composites, and carbon based nanomaterials in DSSCs has increased considerably^11–22^. The carbon based nanomaterials such as graphene, carbon nanotubes (CNTs), and carbon nanoparticles have shown tremendous potential for the development of DSSCs owing to their unique electrical and mechanical properties^23,24^. Previous reports have shown that incorporation of carbon nanomaterials such as reduced graphene oxide, even in natural dye based DSSC can significantly help in improving its efficiency^25^.

On the other hand, storing the electrical energy generated by the DSSCs is another important task to meet the regular energy demands, owing to the irregular nature of power generation. For instance, the solar power cannot be produced at night; however, it is important to supply the electrical energy at night as well in accordance with the demand, which can be accomplished only by storing the electrical energy produced at day. Supercapacitor is a promising energy storage device with high energy density, high charge/discharge rates, and long cycle life performance^26,27^. Supercapacitors have also the potential to replace batteries in near future for the advanced energy storage applications^28^. Supercapacitors are typically used either to accompaniment or to replace batteries in applications that require specific energy densities below 20 Wh/kg and high specific power densities above 10 kW/kg^29^. However, it is still challenged for supercapacitors to match with the lithium ion batteries in terms of energy density^30^. Supercapacitors can be divided into two types based on the charge storage mechanism, viz., Electric double layer capacitors (EDLCs) and Pseudocapacitors^31^. In EDLCs, the charge is stored electrostatically at the Helmholtz layers between electrode–electrolytic ions. Therefore, the specific capacitance of the device can be enhanced by improving the effective electrode surface area accessible by the ions, thereby enhancing the quantum capacitance and the electrical conductivity of the electrode^32^. On the other hand, pseudocapacitors usually exhibit the higher specific capacitance and energy density as compared to the EDLCs due to the electrochemical nature of the charge storage through faradic charge transfer between the electrode and electrolytic ions^33,34^.

Typically, metal oxides like Fe_3_O_4_, MnO_2_, RuO_2_ and V_2_O_5_ were used as active layer materials in pseudocapacitors. However, the pseudocapacitive electrodes show poor electrical conductivity due to the restriction of Faradic reactions because of inferior stability and durability, thereby disappointing electrochemical performance and life cycle^35^. Graphene, a 2D nanomaterial of the carbon family has demonstrated extraordinary electrical, mechanical and optical properties, and proved to be a great material for energy conversion and storage applications such as fuel cells, solar cells and supercapacitors^36–39^. In particular, graphene can offer an enormous specific capacitance of ~ 550 F/g, if the whole surface area of 2630 m^2^/g is utilized^40,41^. In recent years, various studies have used graphene and its composites made of transition metal oxides/hydroxide nanoparticles and conductive as well as non-conductive polymers as electrodes to enhance the capacitance of supercapacitors. Ruoff et al*.* synthesized a chemically modified graphene for supercapacitor electrodes, and fabricated the devices with aqueous and organic electrolytes, achieving a specific capacitance of 135 and 99 F/g, respectively^42^. Yang et al*.* reported the graphene-MnO_2_ composite synthesized through the hydrothermal process and obtained a high specific capacitance of 211.5 F/g at the potential scan rate of 2 mV/s with 1 M Na_2_SO_4_ electrolyte^43^. Park et al*.* investigated graphene modified with nafion as electrode material and obtained a specific capacitance of 118.5 F/g, which is twice that of pristine reduced graphene oxide film electrodes^44^. Recently, Li et al*.* reported the porous graphene paper electrode based supercapacitor with a specific capacitance of 100 F/g at the scan rate of 100 mV/s^45^. The methods generally employed for the synthesis of GNs include mechanical exfoliation, electrochemical exfoliation, chemical exfoliation of graphite powder, chemical vapour deposition (CVD) on the metal surfaces, epitaxial growth on single crystal SiC, chemical coupling reactions, and intercalation/sonication^46,47^.

**Figure 1:**
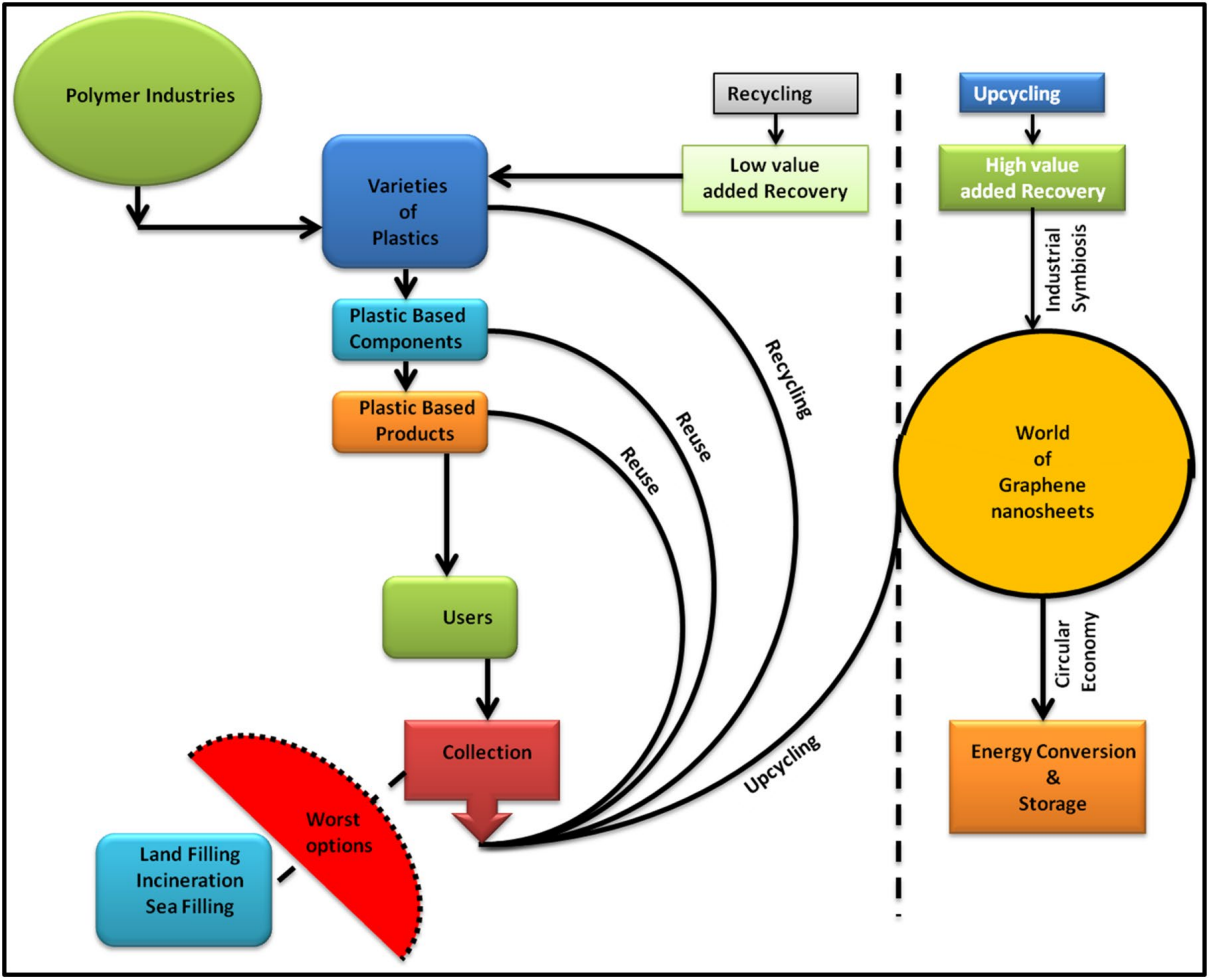
Process flow chart for the waste plastic derived options for circular economy through industrial symbiosis.

However, none of these methods offer convenience for the mass production of GNs. While the cost-effective methods are necessary to deal with the synthesis of carbon nanomaterials, the abundance of precursor material is also important for its mass production. Waste materials containing organic moiety are the best choices to be used as precursors for the mass production of carbon nanomaterials. Fortunately, the high carbon content of plastic waste offers an opportunity to use them as a source or precursor for the synthesis of carbon nanomaterials. Although, waste plastic management has become a contemporary issue for every nation on earth due to the ever increasing utilization and disposal of the plastics. The thermal pyrrolysis of waste plastics in presence of various catalysts to get graphitic nano products can enable the value added recovery of the polymeric products in cost effective manner. Among the variety of plastic products, polypropylene (PP), polyethylene (PE), and polystyrene (PS) are considered to be good precursor materials for the synthesis of carbon nanomaterials due to their high carbon content. Several studies have been done to synthesize the carbon nanomaterials such as CNTs, carbon spheres, carbon nanofibres, graphene nanosheets (GNs) from the plastic waste^48–56^. Thus, the potential value added recovery offers interesting possibilities from economic and environmental perspective in comparison to the traditional high cost recycling. Therefore, if the industries related to graphitic nano products and fuels implement this technology, it could lead to a revolution by offering the following advantages, (1) reduction in costs associated with the management of plastic waste, (2) improved supply of raw material needed to produce graphitic nanomaterials, (3) minimizing the harm to the mother nature through elimination of land fillings and (4) high profits to the industries due to the value added recovery. Figure 1 showed the process flow chart for the waste plastics to get industrial symbiosis and circular economy for the conservation of EEE i.e., ecology, economy and energy.

**Scheme 1:**
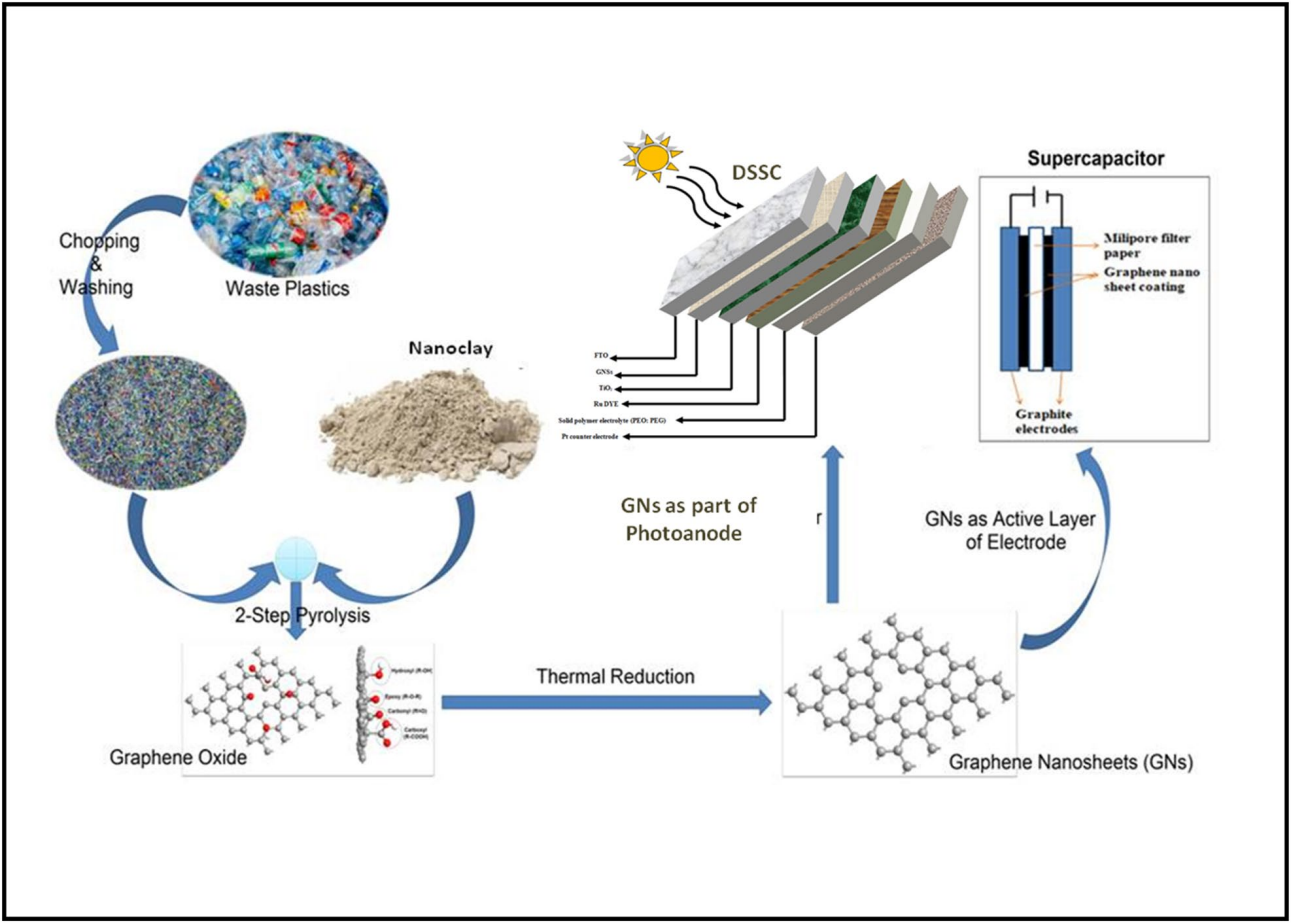
Schematic of the present process depicting the synthesis procedure of GNs from waste plastics and their applications for DSSC and supercapacitors.

Recently, our group reported a very cost effective and facile method for the bulk synthesis of high-quality GNs from plastic waste via two-step pyrolytic procedure^55^. This method of converting the plastic waste into GNs shows the prominence of upcyclization for the conservation of ecology and economy at the same time. In the present work, we report the direct application of reduced GNs produced from the waste plastics for DSSC utilizing solid polymer electrolyte for maximum stability. The device thus fabricated shows optimum performance for DSSCs applications. Moreover, the reduced GNs produced from the waste plastics are also used to fabricate the supercapacitor device (Scheme 1). The results suggest that GNs active material based supercapacitor shows promising capacitance for energy storage applications. Furthermore, computational analysis has also been performed through Density Functional Theory (DFT) based first-principles simulations to predict the suitability of defected GNs over GO and pristine graphene as supercapacitor electrode, and DSSC photoanode as well as absorbance enhancer. The computational results are in line with our experimentations where the defected GNs obtained from waste plastics have been utilized to design high performance Supercapacitors and DSSCs. Furthermore, the computational results suggest that reducing the GO to obtain RGO or defected GNs is the right approach to design high performance supercapacitors and DSSCs, which was followed in our experimentations. The cost benefit analysis shows that the upcyclization of waste plastics into GNs and their subsequent utilization in energy conversion and storage devices could lead to revolutionary changes for the sustainable management of ecology and economy.

## Results and discussions

**Figure 2:**
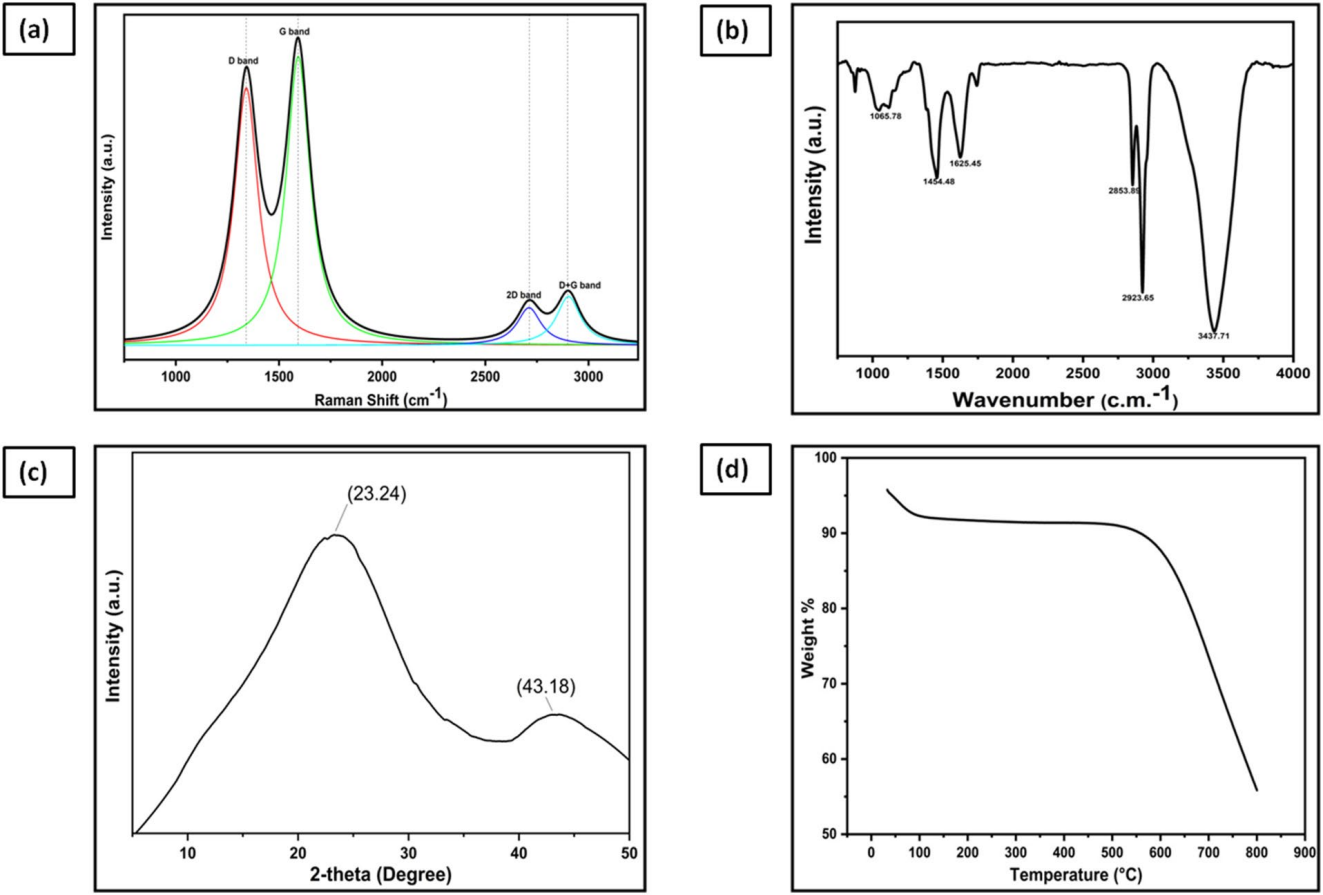
(**a**) Raman spectra of GNs, (**b**) FT-IR spectra of GNs, (**c**) XRD spectra of GNs, (**d**) TGA graph of GNs.

In order to demonstrate the usefulness of GNs prepared from the waste plastics through industrial symbiosis manner, the energy conversion and storage devices namely DSSCs and supercapacitors have been fabricated. The obtained GNs from our pilot scale plant as per the previously reported process^55^ have been thermally reduced at higher temperature (945 °C) before utilizing for these applications. The reduced GNs were critically analyzed by Raman spectroscopy in order to confirm the uniformity of GNs, which is essential for the determination of their purity level before utilizing as photoanode layer in DSSCs and active layer material in supercapacitors. The GNs showed two prominent peaks at 1340/cm and 1591/cm corresponded to D and G bands, respectively (Fig. 2a). The D band demonstrated the stretching of sp^3^ carbon atoms, while the G band showed the stretching of sp^2^ carbon atoms. As reported earlier, during the synthesis process, some of the sp^2^ carbon atoms get distorted and converted to sp^3^ carbon atoms due to the presence of various functional groups as confirmed by the FT-IR spectroscopy. Therefore, the distorted geometry at some of the carbon atoms was confirmed by the presence of D band. The other carbon atoms which possess sp^2^ hybridization, resulted a degree of graphitization in the GNs. Further, the intensity of the D band gives information about the domains of sp^2^ carbons within the GNs, while the average size of the domains of sp^2^ carbons is depicted by the I_D_/I_G_ ratio^57–60^. The I_D_/I_G_ ratio for the GNs was found to be 0.91, thereby showing more domains of sp^2^ carbons within the reduced form of GNs. Additionally, the peaks at 2712/cm and 2896/cm depicted the presence of 2D and D + G bands due to process of double resonance. The 2D band demonstrated the presence of few layer graphene sheets within GNs, which was again confirmed by the I_2D_/I_G_ ratio (0.23). The presence of the high intensity D + G band showed low disorderness of GNs. Further, higher domains of sp^2^ carbon atoms within the GNs are necessary to achieve high performance in DSSCs and supercapacitor devices. In this case, the thermal reduction of GNs enhanced the domains of sp^2^ carbon atoms by reducing oxygen content within GNs as suggested by the EDX analysis. The EDX analysis showed that the GNs were constituted of only carbon and oxygen elements with the wt% of 98.24% and 1.76%, respectively, suggested the high level of purity after thermal reduction, whereas the same as per our previous report was 76.99% and 21.1% for unreduced GNs^53^. From this observation, it was concluded that the treatment of GNs at 945 °C reduced the GNs and hence enhanced the domains of sp^2^ carbon atoms. However, due to the presence of some strongly bonded oxygen functionalities, the FT-IR spectra showed carbon–oxygen, carbon–oxygen–carbon in epoxy ring and combination of O–H deformation at 1065/cm, 1454/cm, and 3437/cm, respectively. The FT-IR spectra also depicted the presence of aromatic double bond and C–H in plane deformation vibrations at 1625/cm, 2853/cm, 2923/cm (Fig. 2b). Moreover, the thermal reduction also reduced the sheet resistance from 400 to 6 Ω/sq, thereby enhancing their suitability as photoanode layer of DSSCs and active layer material of supercapacitors.

The XRD analysis was also performed on the synthesized GNs (Fig. 2c). The XRD spectrum showed diffraction peaks at 2θ = 23.24 and 43.8° corresponded to the characteristic peak and graphitic peak of the reduced GNs. The thickness measurement and evaluation of number of layers within the GNs were done using the XRD graph, as reported previously^61^. The number of layers within GNs can be evaluated by dividing the crystal size (c) with the interlayer distance (d) followed by the addition of thickness of one graphene sheet (0.1 nm). The crystal size (C) was calculated by using Scherer equation^62^, while the Bragg’s equation was used to calculate the interlayer distance (d) within the GNs^63^. The calculated crystal size of the GNs against the diffraction peak at 2θ = 23.24 was found to be 7.22 Å. The interlayer distance (d) was found to be 3.96 Å. Thus according to the XRD data, about 2–3 graphene layers were found within the waste plastic derived GNs. The calculated thickness of GNs from the XRD analysis shows resemblance with the thickness analyzed from the HRTEM images (Fig. 6), which corresponded to the functionalized graphene sheets with few oxygen-containing groups. Therefore, FT-IR investigation was carried out to reflect the presence of the various functional groups within the GNs (Fig. 2c). Further, the presence of these volatile groups and the purity of the graphitic nature of the GNs were evaluated by thermogravimetric analysis (TGA). TGA analysis is one of the few important tools to understand the skeleton properties of GNs as the FT-IR spectra indicated the presence of various groups (Fig. 2d). TGA graph depicted the weight profile of reduced GNs powder w.r.t the variation of temperature (heating rate 1°/min) in presence of N_2_ flow. A relatively small amount of weight loss (~ 3.4 wt%) was observed in the temperature range 35–100 °C corresponded to the water molecules present within the sample, thereafter a clear single step degradation pattern can be seen due to the presence of very small amount of oxygen within the GNs (1.54%, EDX analysis), which shows high purity level of the thermally reduced GNs.

In our previous work^55^, we reported that the defected graphene is more suitable than the pristine graphene for energy storage applications such as battery electrodes, supercapacitor electrodes, etc. In this work, DFT simulations have been performed to show how our thermally reduced defected GNs are a great choice over the unreduced GO and the pristine GNs for application as semiconducting layer in DSSCs and electrode active layer material in supercapacitors.

**Figure 3:**
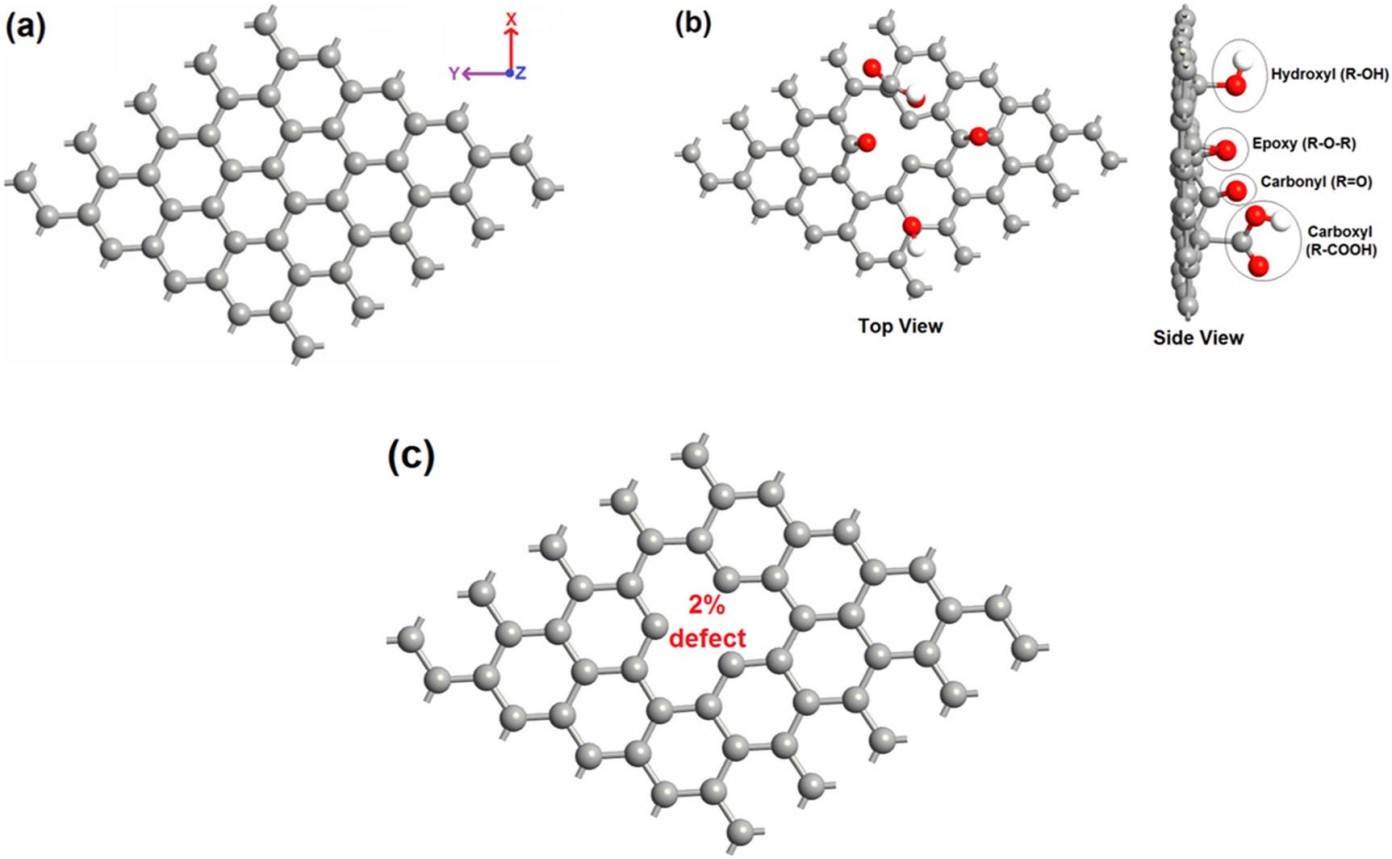
(**a**) 5 × 5 hexagonal periodic supercell of pristine GNs. (**b**) Defected GO with defect concentration of 2%. (**c**) Defected GNs with defect concentration of 2%.

The simulations have been carried out using Synopsys-QuantumATK, a commercial DFT code^64^. The exchange and correlation interaction energy of electrons has been described using the revised Perdew–Burke–Ernzerhof (rPBE) functional within the Generalized Gradient Approximation (GGA) method^65^, while extracting the structural and electronic properties; whereas the optical absorption spectra have been extracted using semi-empirical Meta-GGA (MGGA) functional of *Tran and Blaha*^66^. We have refrained from using MGGA to extract the structural and electronic properties of our graphene structures due to its inability in accurately estimating the total energies and band structures of metallic natured materials. The Kohn–Sham equations are solved by expanding the wave functions using localized Double-Zeta-Polarized (DZP) basis sets. A large density mesh cutoff of 130 Hartree has been used for accuracy of calculations. The Brillouin zone of 5 × 5 hexagonal supercell (consists 50 atoms) has been sampled using Monkhorst–Pack grid of 10 × 10 × 1 k-points, whereas the density of states (DOS) have been extracted using high k-points of 27 × 27 × 1 for accuracy. Computationally, both the defected GO and defected GNs are designed from the pristine GNs of 5 × 5 hexagonal supercell. The defected GNs of 2% and 6% concentration are obtained by subjecting the pristine GNs to single and triple vacancy defects, respectively; whereas the defected GO sheets are obtained by subjecting the pristine GNs to vacancy defects as well as subsequent oxidization with various functional groups viz. epoxy (R–O–R), carbonyl (R=O), hydroxyl (R–OH), and carboxyl (R–COOH). All these structures have been optimized with the help of limited-memory Broyden–Fletcher–Goldfarb–Shanno (L-BFGS) quasi-Newton method^67^, so that the forces on the atoms and stress on the supercell converge below the tolerance value of 0.05 eV/Å and 0.0006 eV/Å^3^, respectively. Figure 3a–c shows the optimized structures of pristine GNs, defected GO and defected GNs with a defect concentration of 2%.

**Figure 4:**
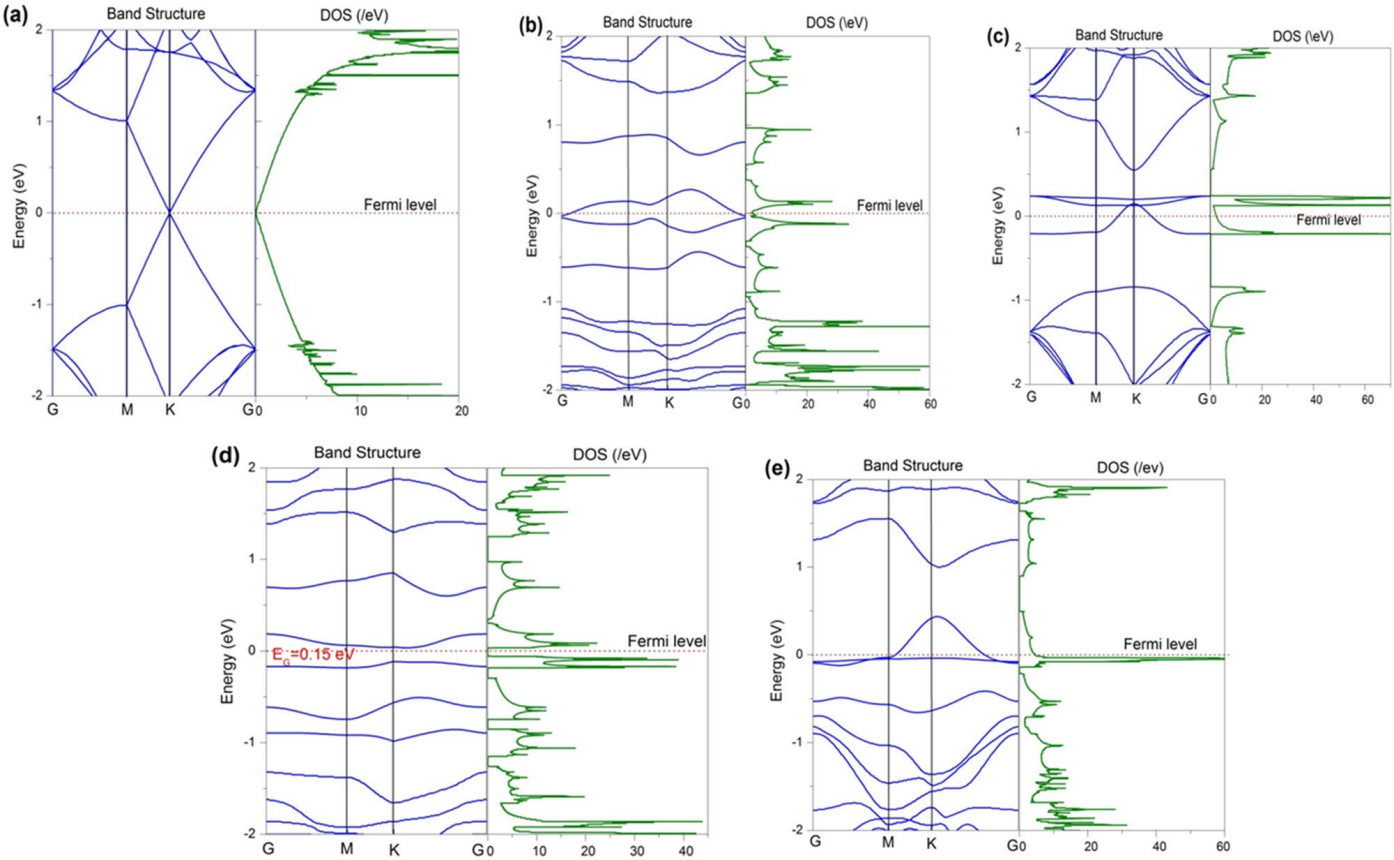
Band structure and DOS profiles for (**a**) pristine GNs, (**b**) 2% defected GO, (**c**) 2% defected GNs, (**d**) 6% defected GO, and (**e**) 6% defected GNs.

The structural properties of the graphene structures have been assessed with the help of C–C bond lengths and thermodynamic stability, as tabulated in Table 1. The pristine GNs has a C–C bond length of 1.42 Å, whereas the same for defected GO and defected GNs varied between 1.35 and 1.63 Å and 1.37–1.51 Å, respectively. The defected GO experienced large variation in C–C bond lengths than the defected GNs, and the maximum C–C bond length of the former reached upto 1.63 Å. This large increment in bond length and the slight out of plane alignment of the carbon atoms associated with the functional groups signify the degree of *sp*^*3*^ hybridization present in the defected GO. On the other hand, the pristine and defected GNs remain purely *sp*^*2*^ hybridized as per our computational investigation. The thermodynamic stability of these sheets is assessed with the help of formation energy calculated per unit length of the sheet (E_F_^L^), as shown in Eq. (1)^68^,

1 $$ E_{F}^{L} = \frac{{\left[ {E_{defected\;GNs/GO} - x*E_{Carbon} - E_{functional\;groups} } \right]}}{L} $$

here, E_defected GNs/GO_, E_carbon_, and E_functional groups_ refer to the energy of defected GNs/GO, energy of single carbon atom of pristine graphene, and energy of functional groups (epoxy, carbonyl, hydroxy, and carboxy), respectively. L and x indicate length of the sheet and the number of carbon atoms present in the sheet, respectively. Table 1 shows the calculated formation energies for defected GNs/GO, where the lower formation energy indicates better thermodynamic stability. Moreover, the positive (negative) sign against the formation energy indicates endothermic (exothermic) process, where energy is consumed (released). It is worth noting that the formation of defected graphene from pristine graphene is an endothermic process, whereas the formation of defected GO from pristine graphene is an exothermic process. In general, the exothermic processes are energetically more favorable than the endothermic processes. This may be the reason that the bulk synthesis of graphene sheets yield GO first, rather than rGO or defected GNs directly, as witnessed in our previous study^55^. Furthermore, the formation energy increases with the defect size as seen from the Table 1, indicating the reduced stability of the defected GNs/GO with increasing defect size. In order to estimate the electronic nature of these sheets, the band structure and density of states (DOS) have been extracted and plotted in Fig. 4.

**Table 1:** Carbon to carbon (C–C) bond length, formation energy per unit length (E_F_^L^), electronic nature, peak quantum capacitance (C_Q_), and the peak absorption coefficient (α) for various graphene/GO structures.

Structure type	C–C bond length (Å)	E_F_^L^ (eV/Å)	Electronic nature	Peak C_Q_ (μF/cm^2^)	Peak α (/nm)
Pristine GNs	1.42	–	Zero band gap	10.73	0.035 (XX, YY) (UV)
2% defected GO	1.37–1.63	− 0.57	Metallic	100.23	0.021 (XX) (IR), 0.018 (YY) (IR)
2% defected GNs	1.40–1.45	0.66	Metallic	185.25	0.02 (XX, YY) (Vis)
6% defected GO	1.35–1.59	− 0.45	0.15 eV	116.01	0.017 (XX) (IR), 0.004 (YY) (IR)
6% defected GNs	1.37–1.51	0.93	Metallic	252.87	0.023 (YY) (Vis), 0.012 (XX) (Vis)

Here, XX and YY indicate zig–zag and armchair directions of the sheets, respectively. UV, IR and Vis indicate the Ultra Violet, Infra Red and Visible regions of the electromagnetic spectrum, respectively, where the peaks have been witnessed in the absorption spectra.

From Fig. 4a, the pristine graphene exhibits a perfect zero band gap with the Dirac cones meeting at K-point of Brillouin zone. At 2% defect concentration, both the defected GO and defected GNs exhibited metallic nature, with the latter possessing more DOS near the Fermi level than the former (Fig. 4b,c). When the defect concentration is increased to 6%, defected GO exhibited a band gap of 0.15 eV, whereas the defected GNs retained its metallic nature (Fig. 4d,e). We have also checked the electronic nature by increasing the defect concentration. Our results suggest that the defected GNs mostly preserves its metallic nature and is thus preferable as electrode material over GO and pristine GNs. The experimental observation of enormous reduction in the sheet resistance after thermal reduction of oxidized GNs may be attributed to the variation in electronic nature, as observed computationally.

**Figure 5:**
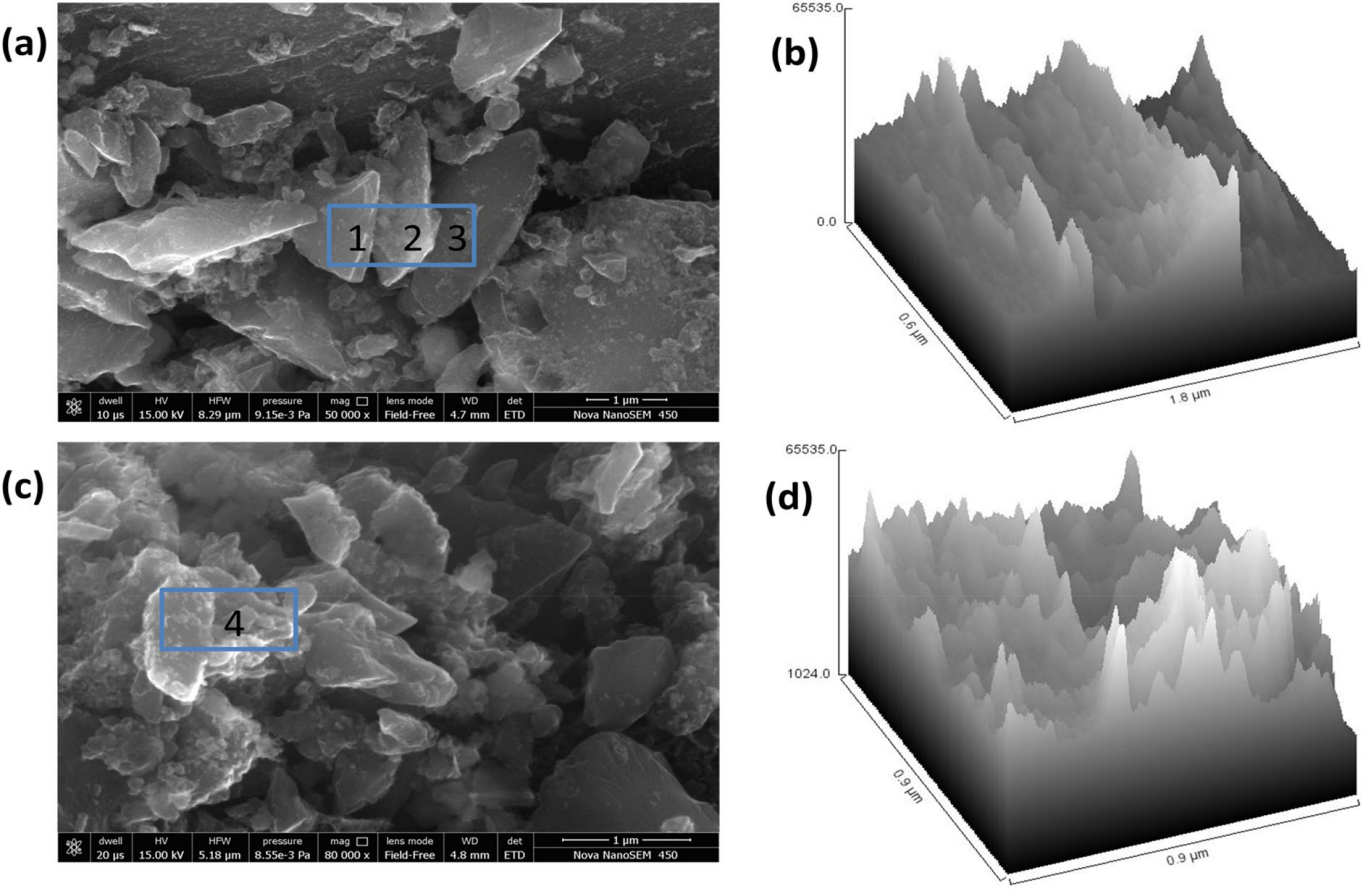
(**a**,**c**) FESEM image of GNs, (**b**,**d**) Hill stack plot of GNs.

Further, the field emission scanning electron microscopy (FESEM) images show a clear rough and porous morphology of the GNs, which postulate the metallic nature of synthesized GNs. The FESEM images in Fig. 5 clearly show large clusters of the graphitic sheets with the presence of wrinkles at the surfaces. Figure 5a shows three consecutive stacks of few layer GNs designated as 1, 2 and 3, which indicate the surface morphology of GNs. The 3D surface morphology of these randomly selected stacked GNs indicate the varying area of individual sheets with corrugated edges (Fig. 5b). Further, these randomly selected GNs stacks are found to be linked with each other by weak Van der Waals forces. Another randomly selected stack of GNs designated by number 4 in Fig. 5c shows the individual sheets of GNs in close proximity, which are held by weak Van der Waals forces. The inflated form of the GNs in randomly selected stack may be a result of thermal treatment during the reduction process. This can be seen in the 3D surface morphology of the selected stack in Fig. 5d, where the sheets show significant separation among them with corrugated edges.

**Figure 6:**
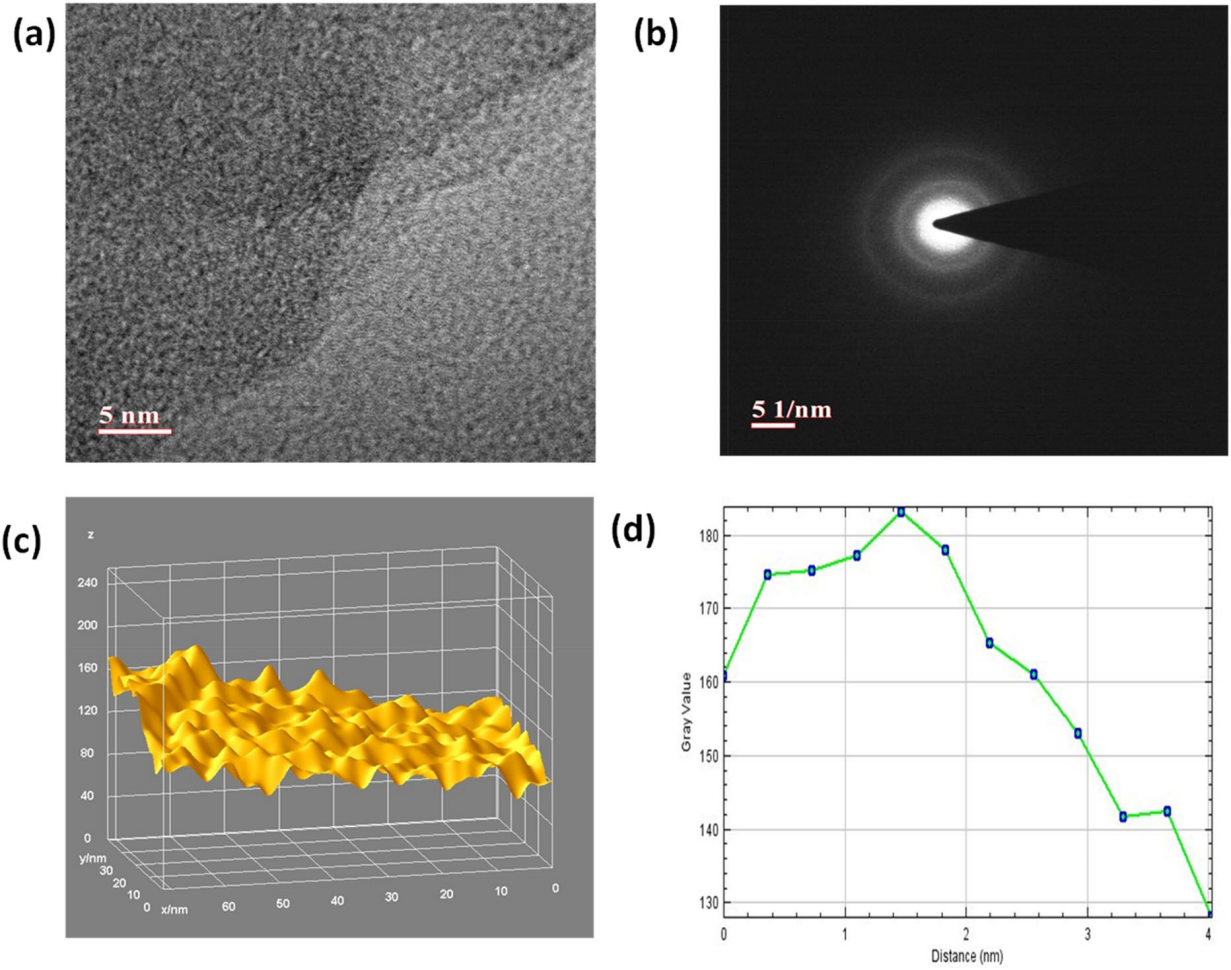
(**a**) HRTEM images of GNs at the magnification of 5 nm, (**b**) SAED pattern for GNs, (**c**) 3D surface plot of HRTEM image of GNs; (**d**) Plot profile diagram for showing the average number sheets present within GNs.

The identification of number of layers in each cluster is found to be difficult from FESEM images, therefore HRTEM analysis was performed in order to get the average number of layers and crystalline behavior of the reduced GNs. Figure 6a,b shows the HRTEM image at a magnification of 5 nm and selected area electron diffraction (SAED) patterns for GNs, respectively. Figure 6a gives a clear view of different kinds of grain boundaries, thereby portraying poly crystalline properties within the GNs. It was again confirmed by selected area electron diffraction (SAED) patterns of GNs (Fig. 6b), where the diffraction patterns in the concentrated circle forms confirm the polycrystalline properties within the GNs. Further, the HRTEM image of the GNs clearly depicted the presence of the 3–5 layers, while the average number of sheets in the GNs were found to be below 5 as depicted by Fig. 6d. This shows the high quality of GNs and their suitability for DSSC and supercapacitor applications. Further, the 3D HRTEM image of the GNs show corrugated edges (Fig. 6c), which makes them favorable for the DSSCs and supercapacitors as per our previously reported work^53^.

After the successful assessment of the synthesis, thermal reduction, and structural as well as electronic aspects of the GNs derived from waste plastics, they have been used to fabricate the DSSCs and for energy conversion and storage purposes.

### Assessment of GNs as a part of the photo-anode layer of DSSC

The suitability of the defected GNs as *a part of the photo-anode layer* and efficient photon absorber^69^ for DSSC has been evaluated with the help of optical absorption spectra, and compared with the defected GO and pristine graphene. The optical absorption spectrum has been calculated from the frequency dependent complex dielectric constant, which has been computed through the Kubo–Greenwood formalism for calculating susceptibility tensor as^70^,

2 $$\varepsilon \left(\omega \right)=(1+{\chi }_{ij}(\omega ))$$

and,

3 $$ \chi_{{{\text{ij}}}} \left( \omega \right) = \frac{{ - e^{2} \hbar^{4} }}{{m^{2} \varepsilon_{0} V\omega^{2} }}\sum\limits_{nm} {\frac{{f\left( {E_{m} } \right) - f\left( {E_{n} } \right)}}{{E_{nm} - \hbar \omega - i\hbar \Gamma }}} \pi_{nm}^{i} \pi_{mn}^{j} $$

here, ħ, V, Γ, and ƒ refer to reduced Planck constant, volume, broadening, and Fermi function, respectively. ${\pi }_{nm}^{i}$ is the i-component of dipole matrix element between ‘n’ and ‘m’ states.

The absorption coefficient (α) has been calculated using the real part (ε_1_) and imaginary part (ε_2_) of the complex dielectric function $\varepsilon \left(\omega \right)$ as,

4 $$\alpha =2\frac{\omega }{c}\sqrt{\frac{\sqrt{{\varepsilon }_{1}^{2}+{\varepsilon }_{2}^{2}}-{\varepsilon }_{1}}{2}}$$

**Figure 7:**
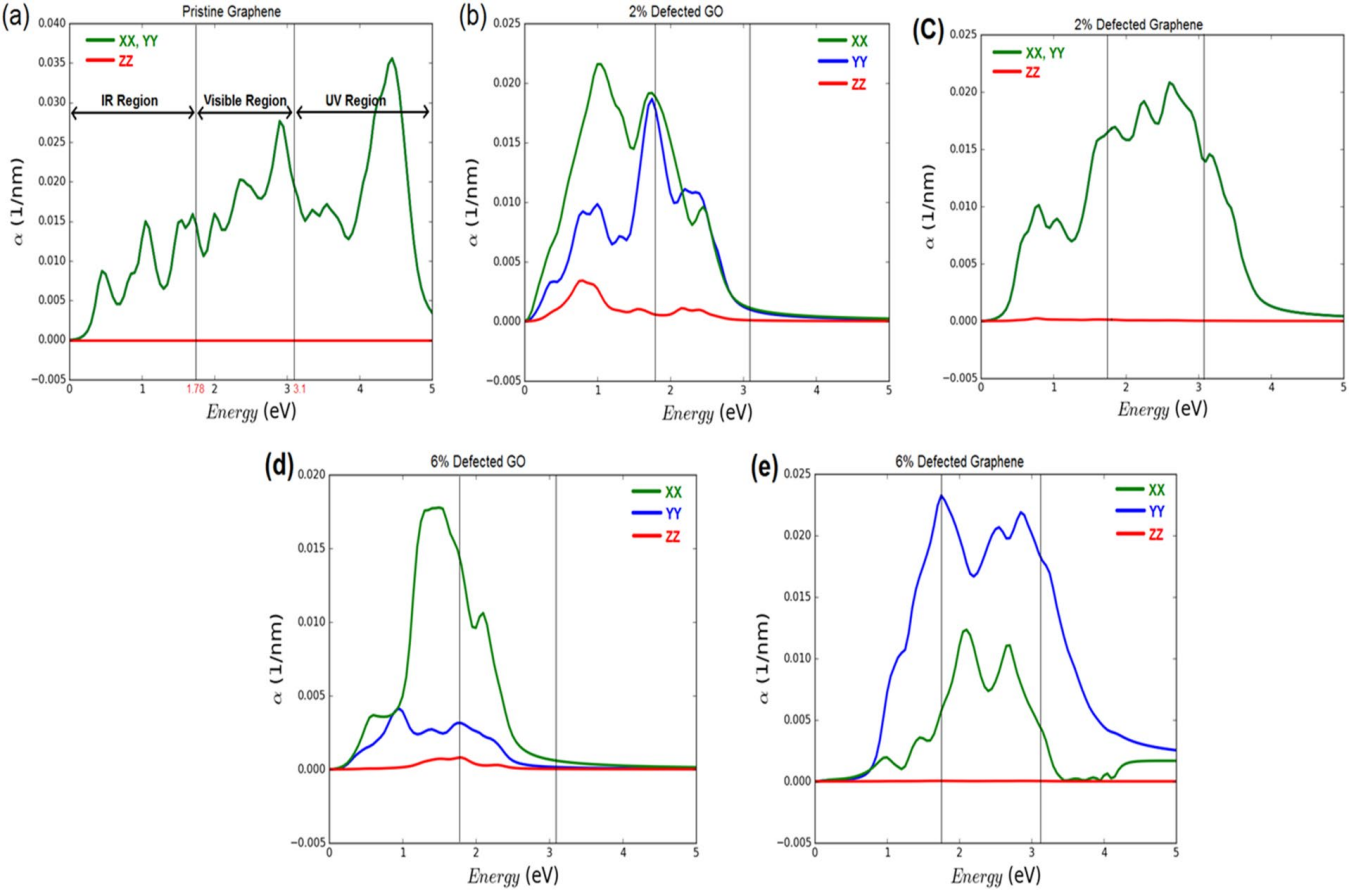
Optical absorption spectra for (**a**) pristine graphene, (**b**) 2% defected GO, (**c**) 2% defected GNs, (**d**) 6% defected GO, and (**e**) 6% defected GNs.

The optical absorption spectra calculated over the photon energy range of 0–5 eV are depicted in Fig. 7, where the infra red (IR), visible, and ultra violet (UV) regions constitute 0–1.78 eV, 1.78–3.1 eV, and 3.1–5 eV, respectively. XX, YY and ZZ indicate the zig–zag, armchair, and out of plane directions of the sheet, respectively.

The peak values of absorption coefficient offered by each of the sheets are listed in Table 1. It can be seen from Fig. 7a–e that only the defected GNs offer maximum absorption peaks in the visible region, whereas the pristine graphene has its maximum peak located in UV region, and the defected GOs have their maximum peaks in the IR region. The maximum peak offered by 2% defected GNs is 0.02/nm (in XX and YY) at 2.6 eV (visible region); while the 6% defected GNs offers 0.023/nm (in YY) at 1.78 eV (Red light of visible region) and 0.012/nm (in XX) at 2.09 eV (visible region). Though the pristine graphene has its maximum peak (0.035/nm) located at 4.45 eV (UV region), it also offers a good peak value (0.027/nm) at 2.9 eV in the visible region. All these absorption peaks of pristine graphene can be attributed to the π–π^*^ transitions^71^. In general, high absorption in the visible region is required in order to attain high efficiency in DSSCs. Thus, the defected GNs are a better choice than the GO and pristine graphene to enhance the photon absorption in DSSCs, owing to its peaks in the visible region. Though the pristine graphene also offers high absorption in the visible region, we could not recommend it owing to the extreme complexity associated with its bulk synthesis.

**Figure 8:**
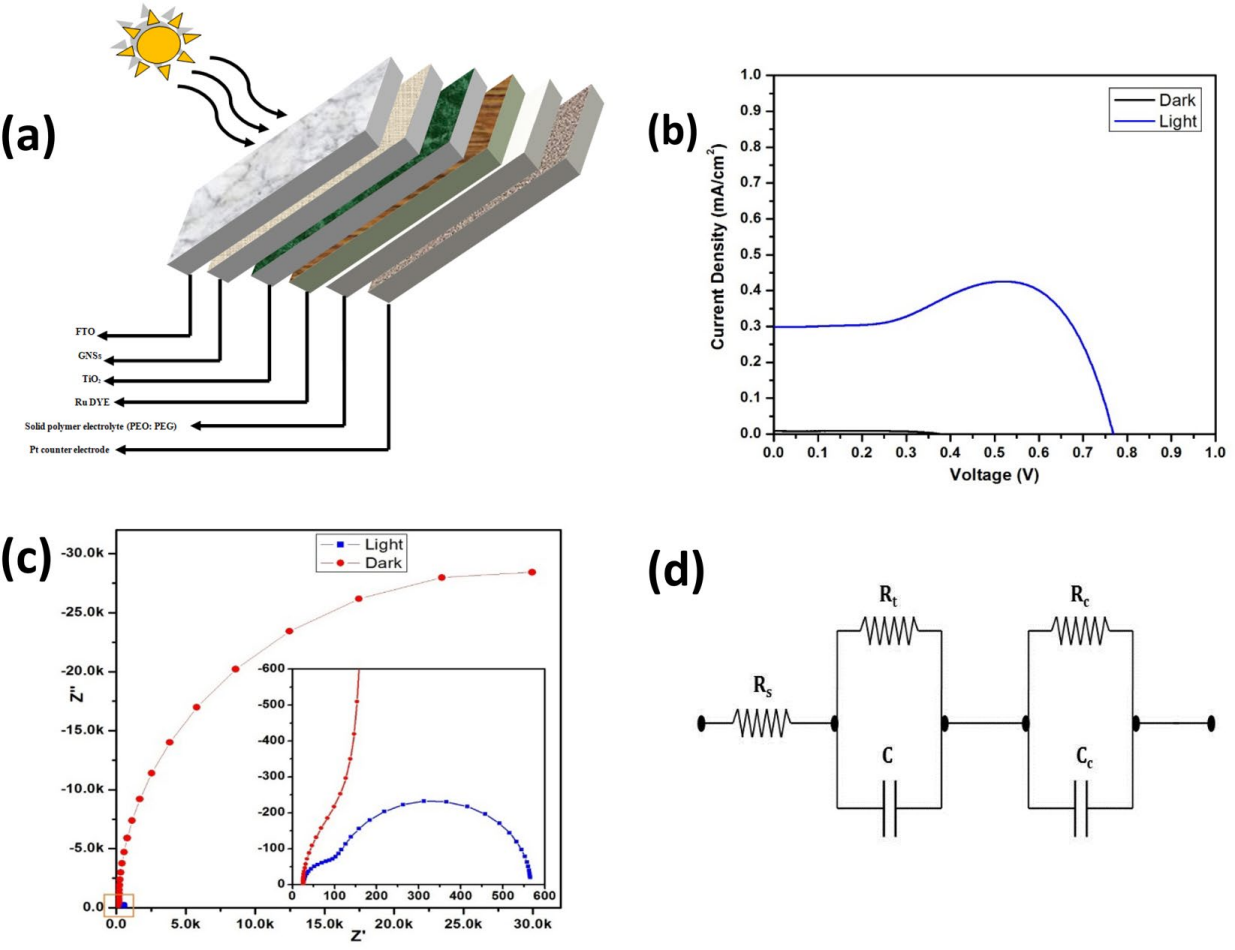
(**a**) Schematic of the fabricated DSSC, (**b**) J–V characteristics, (**c**) EIS curve, and (**d**) Equivalent circuit of the DSSC.

The defected GNs are used to fabricate DSSC, the schematic of which is depicted in Fig. 8a. An area of 0.45 cm^2^ of the fabricated DSSC was exposed to a yellow light emitting halogen lamp (remaining portion was masked with black chart paper) kept at a sufficient height in order to simulate 1 sun condition (100 mW/cm^2^). The Fig. 8b shows the obtained J–V characteristics of the DSSC, which shows almost negligible amount of current under dark conditions, whereas under illumination an open circuit voltage (V_oc_) of 0.77 V with a short circuit current density (J_sc_) of 0.302 mA/cm^2^ has been observed. A gradual rise and fall in the current can be observed between 0.35 and 0.65 V with maxima at ~ 0.5 V, which is a result of the polarization effect of the polymer electrolyte as detailed in a previous study^72^. The obtained fill factor was 86.4% that lead to an overall efficiency (η) of 0.20%. The incorporation of GNs onto the working electrode led to an improvement in the band alignment. Thus, a metallic natured layer between the FTO and TiO_2_ assisted in better collection and transfer of electrons, hence an enhancement in V_oc_.

It has been previously reported that adding an extra layer of GNs in between FTO and TiO_2_ layers can reduce the tendency of charge carriers to recombine by enhancing the charge transport rate and lowering the possibility of recombination, thereby improving the photoelectrical conversion efficiency. Accordingly, under the condition of illumination, the conduction band (CB) of the TiO_2_ received the photoinduced electrons from the photoexcited dye molecules, now as the layer of TiO_2_ is anchored with GNs, the photoexcited electrons were captured rapidly by GNs without any obstacles. Now the collected electrons can transport from TiO_2_ to the conductive FTO substrate more effectively through the bridges of the GNs and hence slowed down the rate of recombination and back reaction^73^. Due to the 2D geometry and porous structure of the GNs, the TiO_2_ nanoparticles anchor well into the GNs, increasing the probability of capturing the photo induced electrons. This results in rapid transportation of the photo induced electrons and suppression of the charge recombination process at the interface. Furthermore, the introduction of GNs into the interface of FTO and TiO_2_ increases the porosity within the system, which enhances the possibility of light scattering during the transmission of incident photons in the device. In other words, incorporation of GNs enhanced the roughness of the interface, which allows more light to scatter and thereby maximizing the device efficiency. The utilization of solid polymer electrolyte enhances the stability of the device in comparison to other reported electrolytes. Further, we have observed that higher concentrations of graphene lowers the efficiency of the system, as the grapheme–TiO_2_ interface that acted as charge collection centre may offer short circuit within the system. In addition to it, the excessive presence of the graphene will also lower the photon absorption characteristics of the dye, thereby reducing the device efficiency. In the present case, we have observed 1.5 wt% of GNs as the optimized parameter for the fabrication of DSSCs.

Furthermore, electrochemical impedance spectroscopy (EIS) was performed using CHI 604D electrochemical workstation for measuring the impedance between the junctions of the cell in both dark and illuminated states. The characteristics obtained after fitting the curve using inbuilt software of the machine is shown in Fig. 8c, and an expanded portion of the squared region is shown in the inset. The equivalent circuit used for the fitting is depicted in Fig. 8d, where R_s_, R_t_, and R_c_ represent the series resistance, the impedance between working electrode and redox electrolyte, and the impedance between counter electrode and electrolyte, respectively. A remarkable difference in the impedance offered by the dark and illuminated conditions is observed. The impedance under illumination is barely visible in comparison to that of dark condition. A much clear observation is made when the graph is expanded to 600 Ω. The value of R_s_ remains 24 Ω for both the conditions as it is the impedance occurring due to the external connections, FTO, etc. The junction impedance between the counter electrode and electrolyte (R_C_) also stays almost the same for both conditions, i.e. 98.33 Ω. The major change is observed in the value of R_t_, which seems to cross 30 kΩ under dark condition, and limited to only 564.83 Ω under illumination.

### Assessment of GNs as active layer material of supercapacitors

The suitability of defected GNs as the active layer material of supercapacitor electrodes has been estimated with the help of quantum capacitance (C_Q_) calculations, and compared with GO and pristine GNs. In general, C_Q_ is the intrinsic capacitance offered by graphene or any other 2D/1D material, and is prominent in those with limited DOS. It plays an important role in determining the total capacitance (C_T_) offered by the graphene supercapacitor, by appearing in series with the double layer or Helmholtz capacitance (C_DL_) that forms between electrode and the electrolytic ions $\left( {{\text{therefore}},\;\frac{1}{{C_{T} }} = \frac{1}{{C_{Q} }} + \frac{1}{{C_{DL} }}} \right)$. Thus, in order to achieve a high total capacitance, it is important to use electrodes that offer high quantum capacitance. Mathematically, C_Q_ can be expressed as the variation caused in excess charge density (Q) of the graphene electrode with respect to the applied potential ($\varphi$), and can be extracted from the DOS profile. The excess charge density on graphene, when the chemical potential is shifted by applying $\varphi /e$, is given by^74^

5 $$Q=e\underset{-\infty }{\overset{\infty }{\int }}D\left(E\right)[f\left(E\right)-f(E-\varphi )]dE$$

here, e, E, D(E), and $f\left(E\right)$ indicate the elementary charge, energy with respect to the Fermi level, DOS, and Fermi–Dirac distribution function, respectively.

Differentiating Q with respect to $\varphi$ yields the differential quantum capacitance,

6 $${C}_{Q}^{diff}=\frac{dQ}{d\varphi }={e}^{2}\underset{-\infty }{\overset{\infty }{\int }}D\left(E\right)[{F}_{T}(E-\varphi )]dE$$

here, the thermal broadening function ${F}_{T}(E)$ is given by,

7 $${F}_{T}(E)=\frac{1}{4KT}{\mathit{sec}h}^{2}\left(\frac{E}{2KT}\right)$$

The C_Q_^diff^ expression that maintains the charge/discharge synchronization between quantum capacitance and DOS profile, is given by,

8 $${C}_{Q}^{diff}=\frac{{e}^{2}}{4KT}\underset{-\infty }{\overset{\infty }{\int }}D\left(E\right){sech}^{2}\left(\frac{E+\varphi }{2KT}\right)dE$$

The integrated quantum capacitance (C_Q_) reported in this work is given by,

9 $$ C_{Q} = \frac{1}{Ve}\int\limits_{0}^{V} {C_{Q}^{diff} \left( {V^{\prime}} \right)dV^{\prime}} $$

**Figure 9:**
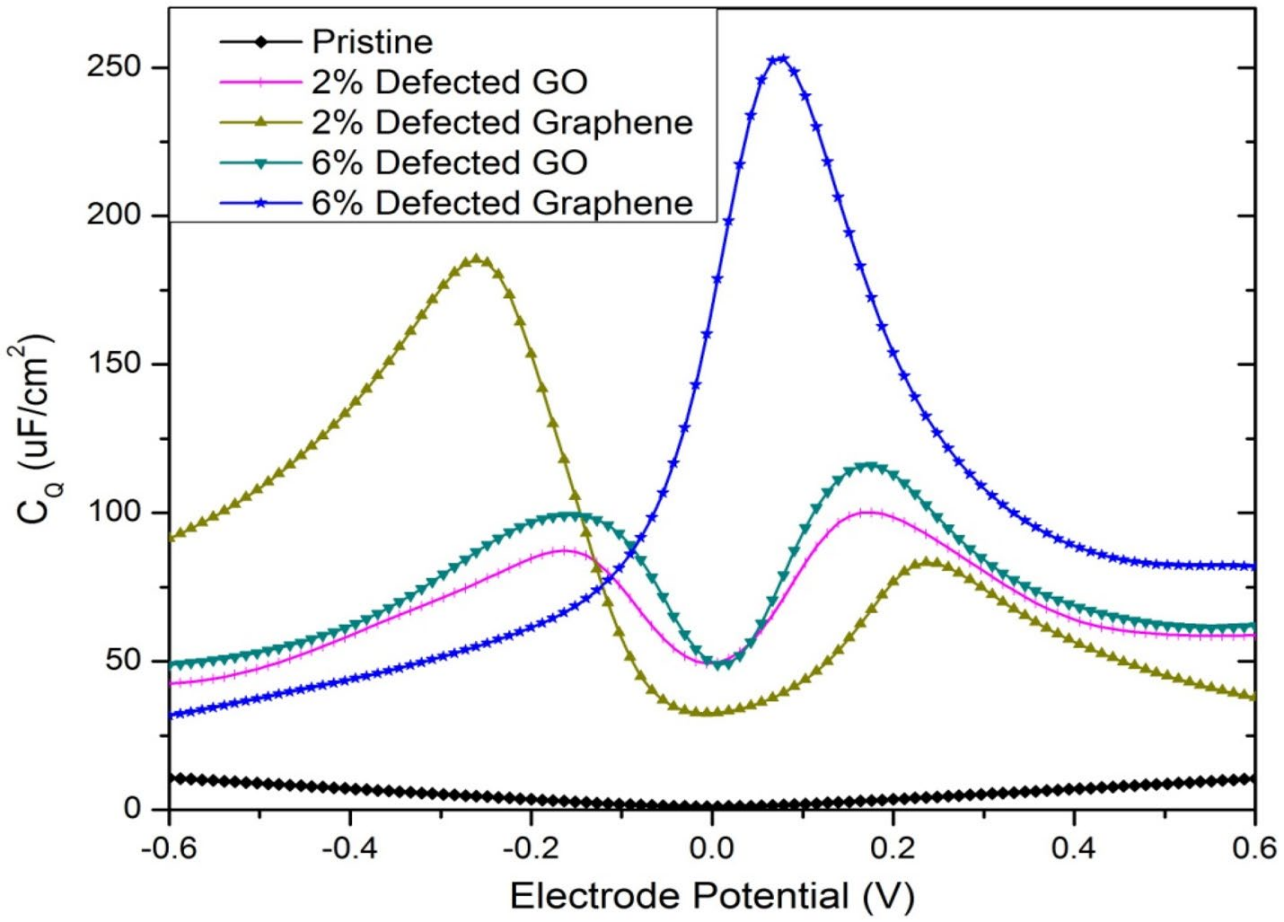
Comparison of quantum capacitance (C_Q_) of defected GNs with defected GO and pristine GNs.

The computed C_Q_ of the defected GNs along with the defected GO and pristine GNs is depicted in Fig. 9, and the peak C_Q_ values are tabulated in Table 1. The C_Q_ is plotted over a potential range of − 0.6 to 0.6 V only, considering the typical electrochemical window of aqueous electrolyte of the supercapacitor. From Fig. 9, pristine GNs posses a very low C_Q_ with the peak value of just 10.73 μF/cm^2^ at − 0.6 V, which is in good agreement with the experimental report by Ponomarenko et al*.*^75^. At a defect concentration of 2%, the defected GNs and the defected GO exhibited peak quantum capacitances of 185.25 μF/cm^2^ and 100.23 μF/cm^2^, respectively. At a defect concentration of 6%, the defected GNs and the defected GO exhibited peak quantum capacitance of 252.87 μF/cm^2^ and 116.01 μF/cm^2^, respectively. Thus, the defected GNs clearly provide superior quantum capacitance over the defected GO and pristine GNs, which makes it an ideal choice for electrode active layer material of supercapacitor devices.

**Figure 10:**
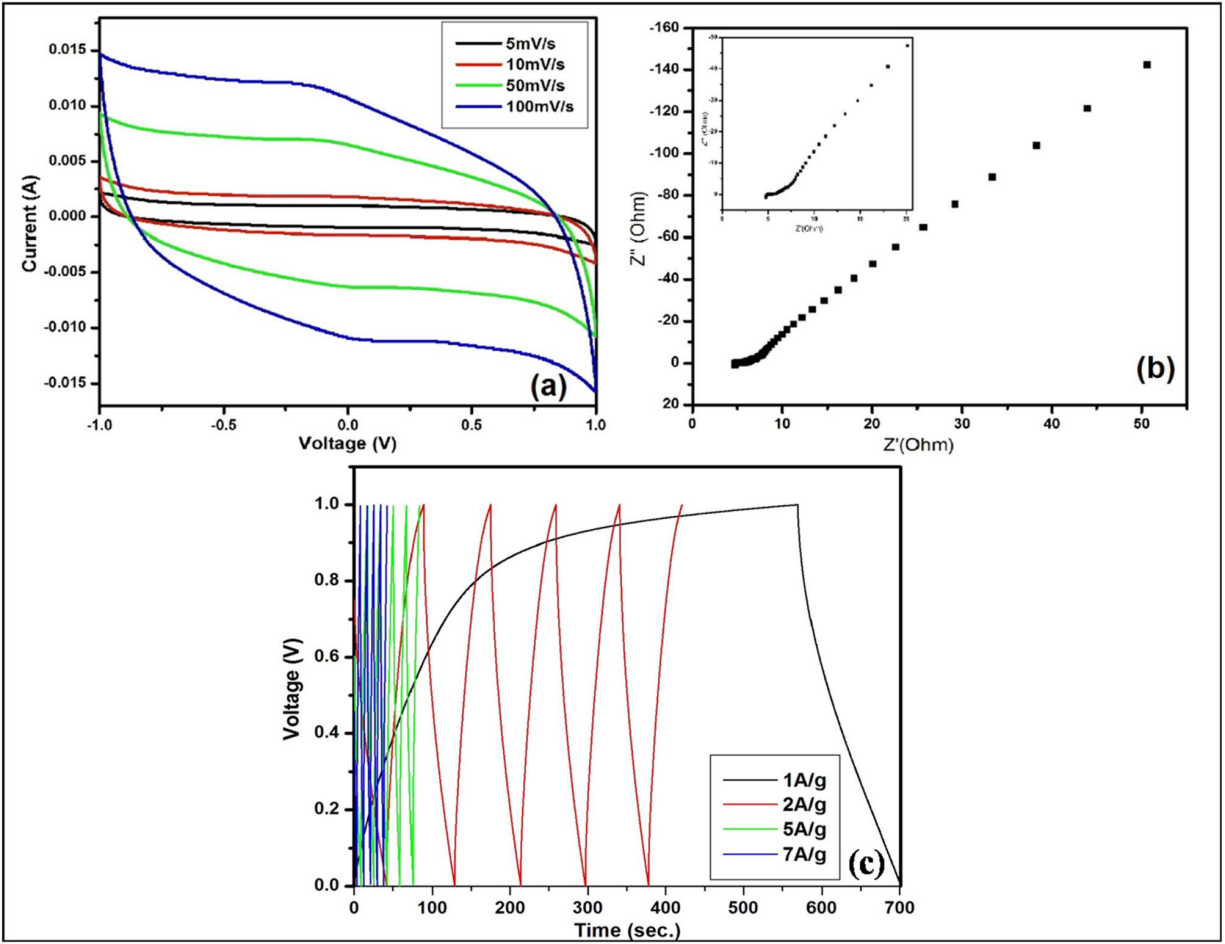
(**a**) CV curve (**b**) EIS spectra (**c**) GCD curve of the GNs based cell in 1 M H_3_PO_4_ electrolyte.

Thus, in order to make a good agreement with computational analysis, waste plastic derived GNs was used as active layer materials for graphite based supercapacitors with a geometry of Graphite sheet|GNs*|*1 M H_3_PO_4_|GNs|Graphite sheet. The defected GNs are mixed with the binder PVDF-HFP (Poly (vinylidene fluoride-*co*-hexafluoropropylene)) in 10% acetone solution for 5–6 h over magnetic stirrer to make sticky paints for the fabrication of the graphite based electrodes. The cyclic voltammetry of fabricated cell (Fig. 10a) showed excellent reversibility over the performed voltage range at the scan rate of 5 mV/s, 10 mV/s, 50 mV/s and 100 mV/s. The CV curve showed nearly rectangular shape due to lower contact resistance and formation of electrical double layers which showed the one of the features for a good supercapacitor. The specific capacitance via CV shown in Table 2 for the fabricated cell is given by

10 $$ {\text{C}} = {\text{ i}}/{\text{s}} $$

where i is the current, s is the rate of the scan. From the CV curve and using the above equation, the fabricated cell showed the highest specific capacitance of 398 F/g at the scan rate of 5 mV/s, while it is found to be 340 F/g, 254 F/g and 215 F/g for the scan rate of 10 mV/s, 50 mV/s and 100 mV/s, respectively.

**Table 2:** Specific capacitance obtained from the cyclic voltammetry analysis at different scan rates.

Cell	Specific capacitance (F/g) at 5 mV/s	Specific capacitance (F/g) at 10 mV/s	Specific capacitance (F/g) at 50 mV/s	Specific capacitance (F/g) at 100 mV/s
Cell	398	340	254	215

Further, EIS of the fabricated cell (Fig. 10b) shown by Nyquist plot were performed to investigate the equivalent sheet resistance (ESR) and specific capacitance at 10 MHz. Figure 10b showed the steeply rising incident toward the lower frequency region which indicates the capacitance behavior shown by the GNs with the electrolytes due to the formation of the electrical double layer at the interface of electrode and electrolyte. The device showed lower ESR value of 4.78 Ω/cm^2^, which showed the sophisticated feasibility of the ion transportation within the GNs. In addition to it, the small semicircle shown by the EIS curve in the higher frequency region indicated the smaller charge transfer resistance, which arises due to the bulk properties of both electrolyte and the contact of GNs and graphite sheet for the fabricated cell. Further, including all the parameter of the EIS study, the specific capacitance for the same cell via EIS curve were calculated by using the following equation

11 $$ {\text{C}} = {1 \mathord{\left/ {\vphantom {1 {\left( {2\pi fZ^{\prime\prime}} \right)}}} \right. \kern-\nulldelimiterspace} {\left( {2\pi f{\text {Z}}^{\prime\prime}} \right)}}$$

where *f* is the frequency of applied AC signal, Z″ is the imaginary part and Z′ is the real part of impedance at 10 MHz. The specific capacitance via this technique and using above equation is found to be 221.77 F/g. Figure 10c shows the galvanostatic charging discharging (GCD) behavior of the cell at 1 A/g, 2 A/g, 5 A/g and 7 A/g of current densities over the potential window of 0–1 V. The cell showed nearly triangular shape of the curve over the performed current densities which arise due to capacitive behavior of the cell. Further, these curves used for the estimation of specific capacitance behavior of the both cell by using the following equation

12 $$ {\text{C}} = {{\left( {{\text{i}}\Delta {\text{t}}} \right)} \mathord{\left/ {\vphantom {{\left( {{\text{i}}\Delta {\text{t}}} \right)} {\left( {{\text{m}}\Delta {\text{V}}} \right)}}} \right. \kern-\nulldelimiterspace} {\left( {{\text{m}}\Delta {\text{V}}} \right)}} $$

where i is the scan rate, m represent the mass loading over the electrode and Δt/ΔV is the linear part slop in the discharge curve. The evaluated specific capacitance has shown in Table 3 for the fabricated cells by using the above equation. The highest specific capacitance value shown by the cell is found to be 273.69 F/g at 1 A/g of current density. Additionally, the fabricated cell shows the very appreciable energy density (E_d_) and power density (P_d_) of 38 Wh/kg and 1009.74 W/kg, respectively which were calculated by using the following equation

13 $$ {\text{E}}_{{\text{d}}} = \, \raise.5ex\hbox{$\scriptstyle 1$}\kern-.1em/ \kern-.15em\lower.25ex\hbox{$\scriptstyle 2$} {\text{ CV}}^{{2}} $$

14 $$ {\text{P}}_{{\text{d}}} = {\text{ E}}/{\text{t}} $$

**Table 3:** The specific capacitance value GCD for the GNs in 1 M H_3_PO_4_ electrolyte.

Cell	Specific capacitance using galvanostatic charge/discharge (F/g) at 1A/g	Specific capacitance using galvanostatic charge/discharge (F/g) at 2A/g	Specific capacitance using galvanostatic charge/discharge (F/g) at 5A/g	Specific capacitance using galvanostatic charge/discharge (F/g) at 7A/g
	273.69	159.95	84.54	59.78

### Supply and environmental impact

The utilization of plastics for various purposes is growing day by day across the globe, and is expected to increase by four fold for every few years. A recent survey on waste plastic generation outlined that around 246 million tons of waste plastic is generated annually. Over the past four decades the waste plastic was increased by four fold, and if not managed properly, it is expected to result in a 15% increase in the emission of green house gases (GHGs)^76^. However, utilizing this waste as a source material to synthesize graphene can establish an uninterrupted supply chain of raw material, and also save the environment from the exponential increment of carbon footprint. It has been estimated based on the previous reports that fabrication of highly efficient graphene based DSSC and supercapacitor of 1 cm^2^ area requires only 1–10 mg of GNs. Therefore, if the waste plastic is efficiently collected and upcycled through the proposed method then the resulting GNs will be sufficient to fulfill the energy conversion, storage, and other applications as per the contemporary market demand.

### Cost benefit assessment

The cost associated with the production of GNs from waste plastic through the proposed industrial symbiosis show a significant 100-fold benefit in comparison to the cost of commercially available GNs. The production cost has been estimated by considering all possible costs which include labor, transportation, electricity, chemicals, sealing, packing and management costs. The cost of producing 10 g of the graphene though our industrial symbiosis process is estimated to be at max 1 USD, whereas the cost of purchasing 10 g of commercially available graphene ranges between 100 and 200 USD. The production cost may further be reduced through bulk synthesis of the GNs and automation of the process. Thus, our proposed method is not only viable but also quite cheaper. To conclude, the conversion of waste plastics into GNs though the proposed method could have dramatic positive impact on both ecology and economy.

## Conclusion

The sustainable environmental protection from the plastic waste could be achieved by diverting this waste to the upcycling processes rather than the traditional recycling processes, as the former offers greater benefit from both the economic and ecologic perspectives. This work presents an industrial symbiosis process for conversion of the waste plastic into grapehene nanosheets (GNs) and their subsequent utilization as a case study for the fabrication of low cost energy conversion and storage devices viz. DSSCs and supercapacitors, respectively. Besides the experimental synthesis and device fabrication, we have also performed the first principle computations to understand and support the experimental results. The DSSC fabricated with GNs as semiconducting layer with polymeric electrolyte exhibited an open circuit voltage (V_oc_) of 0.77 V with a short circuit current density (J_sc_) of 0.302 mA/cm^2^. The fabricated supercapacitor with GNs as active layer of electrode material offered an impressive specific capacitance of 398 F/g with a scan rate of 0.005 V/s. The cost benefit analysis revealed that the proposed upcycling process has the potential to revolutionize waste plastic management and GNs production segments, as the process offers high value added recovery in comparison to the traditional recycling methods, and also produces the GNs about 100 fold cheaper than the cost of acquiring the commercially available GNs.

## Methods

### Synthesis of graphene nanosheets (GNs)

Synthesis of GNs from waste plastics has been performed as per our previously reported procedure^53^. However, in the present work, we have modified our method by changing the temperature range to demonstrate the application of the GNs for DSSCs and supercapacitors. Briefly, waste plastic were collected from local market and categorized into polypropylene (PP), polyethylene (PE) and polyethylene tetrathalate (PET), as these plastic contains maximum amount of carbon in comparison to other kind of plastics. After the categorization, the real world mix plastics (PP, PE and PET) were shredded into the small flakes (average size of 10 mm × 5 mm) with the help of shredder unit, which were later on washed with sodium hydroxide and glass washing soap solution (laboratory grade) to remove all the muddy and oily impurities from the mixed flakes of the plastics. Then the flakes were dried inside the drier chamber connected with hot air gun. Once the mixed flakes of the plastics were dried, mixing of the bentonite nanoclay as degradation agent was done inside the mixing chamber. Inside the mixing chamber, washed and dried mix plastics were mixed with bentonite nanoclay in a ratio of 1000:3 and mixed for 30 min in order to assure uniform mixing of the nanoclay. Here we take 20 kg of the waste plastics and 60 g of the bentonite nanoclay according to the mentioned ratio. After the proper mixing of the bentonite nanoclay with mixed plastics, process of the primary pyrrolysis was performed inside the indigenously developed pyrrolysis chamber in the inert atmosphere of N_2_ with the flow rate of 10 ml/min at the temperature of 450 °C (heating rate 9 °C/min) for 50 min. This step of the pyrolysis removes all the petroleum products while leaving back the carbon skeleton in the form of black charred residue. The black charred residue thus obtained then undergo for the secondary stage pyrolysis process. In our previous work, the synthesis of the graphene nanosheets was depicted at the temperature of 750 °C. However, in the present work we raise the temperature by 945 °C for the reduction of the graphene nanosheets to increase the number of sp^2^ hybridized carbon atoms within the graphene nanosheets, so that these GNs were implemented as a part of photoanode material in DSSC and active layer material in supercapacitor devices, respectively.

### Fabrication of DSSC

The fabrication of DSSC was carried out by following a previously reported method^76,77^. 8 mg of the prepared GNs was added to 4 ml polyethylene glycol (PEG, Sigma Aldrich, Mw = 200) in an eppendorf vial and sonicated for 4–5 days in order to obtain a uniform solution. 50 μl of the prepared solution was spin-coated at ~ 1200 rpm for 60 s on fluorine doped tin oxide (FTO) glass. This was followed by the calcination of FTO glass at 350 °C for 1 h in order to remove the polymer. Two scotch tapes were pasted on both sides of the ‘almost transparent’ layer of GNs for controlling the thickness of the TiO_2_ paste (Alibaba, China). The viscous paste was spread uniformly by doctor blade method followed by calcination at 500 °C for 30 min. The electrode after bringing down to room ambient temperature was immersed in an ethanolic solution of Ruthenium dye (Solaronix, Switzerland) for 6 h. The standard PEO:PEG electrolyte with Iodine redox couple and Platinum coated counter electrode was used for completing the sandwich structure as detailed in previous reports^78^.

### Fabrication of supercapacitors

To fabricate an Electric Double Layer Capacitor (EDLC), we have used 1 cm × 1 cm graphite sheet (current collector) and the GNs as an active layer material with binder PVDF-HFP (Poly (vinylidene fluoride-*co*-hexafluoropropylene)) (Sigma Aldrich, USA) in the ratio (90:10) dissolved in acetone. The GNs was well coated on current collector and kept in laboratory oven at 100 °C overnight. 1 M H_3_PO_4_ with a millipore filter paper (0.45 micron) as separator was sandwiched between the electrodes to perform the Cyclic Voltammetry (CV), galvanostatic charge-discharging (GCD) and Electrochemical Impedance Spectroscopy (EIS) measurements.

### Characterizations

GNs synthesized from waste plastics were characterized by various spectroscopic and imaging techniques as previously reported^53^. However, after thermal reduction of the GNs Raman spectroscopy was performed by using Renishaw Raman Microscope to understand the effect of thermal reduction on the GNs. Furthermore, X-ray diffraction (XRD) analysis was performed by using Rigaku diffractometer with Cu-Kα radiation. FT-IR spectroscopy was performed to analyze the functionality of the GNs by using PerkinElmer spectrum-2. TGA analysis was performed by TGA 4000, Perkin Elmer. Additionally, the surface morphology analysis of the GNs was analyzed through FESEM using NOVA NanoSEM 450. High resolution transmission electron microscopy (HRTEM) was performed by using JEOL, JEM 2100 microscope to get analyze the thickness and crystalline behaviour of the GNs. Sheet resistance of the GNs was analyzed by four probe setup using Keithley 2400 SourceMeter. Current–Voltage characteristics of the fabricated GNs based DSSC was carried out by using Keithley 2400 SourceMeter. Cyclic Voltammetry (CV) analysis, galvanostatic charge–discharging (GCD) and EIS spectroscopy was performed for measuring the impedance between the junctions of the supercapacitor cell by using CHI 604D electrochemical workstation.

## Data Availability

The data that support the plots within this paper and other findings of this study are available from the corresponding author upon reasonable request.

## References

[1] Charles RG (2018). Platinized counter-electrodes for dye-sensitised solar cells from waste thermocouples: A case study for resource efficiency, industrial symbiosis and circular economy. J. Cleaner Prod..

[2] Assessment, Impact. Commission Staff Working Document. in *The Support of Electricity From* (2008).

[3] Mathieux, F., et al. Critical raw materials and the circular economy. in *Publications Office of the European Union: Bruxelles, Belgium* (2017).

[4] MacArthur E (2013). Towards the Circular Economy, Economic and Business Rationale for an Accelerated Transition.

[5] EC. Critical raw materials for the EU. Report of the Ad-hoc Working Group on defining critical raw materials. *Ad-hoc Working Group* 84 (2010).

[6] EC, Speaking points by Environment Commissioner Janez Potočnik on Circular Economy—*Press conference on Circular Economy and Green Employment Initiative, Brussels*. (2014).

[7] EC, Report on critical raw materials for the EU—Critical raw materials profiles. in *Industry, D.E.a.* (ed.) (2015).

[8] Harikisun R, Desilvestro H (2011). Long-term stability of dye solar cells. Sol. Energy.

[9] Baxter JB (2012). Commercialization of dye sensitized solar cells: Present status and future research needs to improve efficiency, stability, and manufacturing. J. Vacuum Sci. Technol. A Vacuum Surf. Films.

[10] De Rossi F, Pontecorvo T, Brown TM (2015). Characterization of photovoltaic devices for indoor light harvesting and customization of flexible dye solar cells to deliver superior efficiency under artificial lighting. Appl. Energy.

[11] Arbab AA (2015). Fabrication of highly electro catalytic active layer of multi walled carbon nanotube/enzyme for Pt-free dye sensitized solar cells. Appl. Surface Sci..

[12] Sahito IA (2016). Flexible and conductive cotton fabric counter electrode coated with graphene nanosheets for high efficiency dye sensitized solar cell. J. Power Sources.

[13] Kim H, Veerappan G, Park JH (2014). Conducting polymer coated non-woven graphite fiber film for dye-sensitized solar cells: Superior Pt-and FTO-free counter electrodes. Electrochim. Acta.

[14] Ahmad S (2010). Dye-sensitized solar cells based on poly (3, 4-ethylenedioxythiophene) counter electrode derived from ionic liquids. J. Mater. Chem..

[15] Lee TH (2012). High-performance dye-sensitized solar cells based on PEDOT nanofibers as an efficient catalytic counter electrode. J. Mater. Chem..

[16] Takada H (2015). Improved durability of dye-sensitized solar cell with H2-reduced carbon counter electrode. J. Power Sources.

[17] Yeh M-H (2014). Multiwalled carbon nanotube@ reduced graphene oxide nanoribbon as the counter electrode for dye-sensitized solar cells. J. Phys. Chem. C.

[18] Chang L-H (2013). A graphene-multi-walled carbon nanotube hybrid supported on fluorinated tin oxide as a counter electrode of dye-sensitized solar cells. J. Power Sources.

[19] Park S-H, Kim B-K, Lee W-J (2013). Electrospun activated carbon nanofibers with hollow core/highly mesoporous shell structure as counter electrodes for dye-sensitized solar cells. J. Power Sources.

[20] Arbab AA (2016). Fabrication of textile fabric counter electrodes using activated charcoal doped multi walled carbon nanotube hybrids for dye sensitized solar cells. J. Mater. Chem. A.

[21] Gong F (2013). NiSe_2_ as an efficient electrocatalyst for a Pt-free counter electrode of dye-sensitized solar cells. Chem. Commun..

[22] Li GR (2011). Highly Pt-like electrocatalytic activity of transition metal nitrides for dye-sensitized solar cells. Energy Environ. Sci..

[23] Yan X (2010). Large, solution-processable graphene quantum dots as light absorbers for photovoltaics. Nano Lett..

[24] Yan X (2011). Independent tuning of the band gap and redox potential of graphene quantum dots. J. Phys. Chem. Lett..

[25] Surana K, Idris MG, Bhattacharya B (2020). Natural dye extraction from Syzygium Cumini and its potential photovoltaic application as economical sensitizer. Appl. Nanosci..

[26] Simon P, Gogotsi Y (2010). Materials for electrochemical capacitors. Nanosci. Technol. Collect. Rev. Nat. J..

[27] Liu C (2010). Advanced materials for energy storage. Adv. Mater..

[28] Kaempgen M (2009). Printable thin film supercapacitors using single-walled carbon nanotubes. Nano Lett..

[29] Miller JR, Simon P (2008). Electrochemical capacitors for energy management. Sci. Mag..

[30] Melot BC, Tarascon JM (2013). Design and preparation of materials for advanced electrochemical storage. Acc. Chem. Res..

[31] Winter M, Brodd RJ (2004). What are batteries, fuel cells, and supercapacitors?. Chem. Rev..

[32] Shi W (2011). Achieving high specific charge capacitances in Fe_3_O_4_/reduced graphene oxide nanocomposites. J. Mater. Chem..

[33] Hu C-C (2006). Design and tailoring of the nanotubular arrayed architecture of hydrous RuO_2_ for next generation supercapacitors. Nano Lett..

[34] Ke Y-F, Tsai D-S, Huang Y-S (2005). Electrochemical capacitors of RuO_2_ nanophase grown on LiNbO 3 (100) and sapphire (0001) substrates. J. Mater. Chem..

[35] Xiao W (2009). Growth of single-crystal α-MnO_2_ nanotubes prepared by a hydrothermal route and their electrochemical properties. J. Power Sources.

[36] Choi H-J (2012). Graphene for energy conversion and storage in fuel cells and supercapacitors. Nano Energy.

[37] Hu K (2014). Graphene-polymer nanocomposites for structural and functional applications. Prog. Polym. Sci..

[38] Punetha VD (2017). Functionalization of carbon nanomaterials for advanced polymer nanocomposites: A comparison study between CNT and graphene. Prog. Polym. Sci..

[39] Novoselov KS (2004). Electric field effect in atomically thin carbon films. Science.

[40] Xia J (2009). Measurement of the quantum capacitance of graphene. Nat. Nanotechnol..

[41] Ke Q, Wang J (2016). Graphene-based materials for supercapacitor electrodes—A review. J. Materiomics.

[42] Stoller MD (2008). Graphene-based ultracapacitors. Nano Lett..

[43] Li Z (2011). Synthesis of hydrothermally reduced graphene/MnO_2_ composites and their electrochemical properties as supercapacitors. J. Power Sources.

[44] Choi BG (2011). Facilitated ion transport in all-solid-state flexible supercapacitors. ACS Nano.

[45] Li Q (2017). Porous graphene paper for supercapacitor applications. J. Mater. Sci. Technol..

[46] Allen MJ, Tung VC, Kaner RB (2010). Honeycomb carbon: A review of graphene. Chem. Rev..

[47] Wu C (2012). Sustainable processing of waste plastics to produce high yield hydrogen-rich synthesis gas and high quality carbon nanotubes. RSC Adv..

[48] Wu C (2014). Processing real-world waste plastics by pyrolysis-reforming for hydrogen and high-value carbon nanotubes. Environ. Sci. Technol..

[49] Yao D (2017). Co-production of hydrogen and carbon nanotubes from catalytic pyrolysis of waste plastics on Ni–Fe bimetallic catalyst. Energy Convers. Manag..

[50] Zhuo C (2010). Synthesis of carbon nanotubes by sequential pyrolysis and combustion of polyethylene. Carbon.

[51] Alves JO (2011). Catalytic conversion of wastes from the bioethanol production into carbon nanomaterials. Appl. Catalysis B Environ..

[52] Ma Q (2017). Carbon-based functional materials derived from waste for water remediation and energy storage. Adv. Mater..

[53] Jiang Z (2007). Polypropylene as a carbon source for the synthesis of multi-walled carbon nanotubes via catalytic combustion. Carbon.

[54] Gong J (2014). Catalytic carbonization of polypropylene into cup-stacked carbon nanotubes with high performances in adsorption of heavy metallic ions and organic dyes. Chem. Eng. J..

[55] Pandey S (2019). Bulk synthesis of graphene nanosheets from plastic waste: An invincible method of solid waste management for better tomorrow. Waste Manag..

[56] Zhuo C, Levendis YA (2014). Upcycling waste plastics into carbon nanomaterials: A review. J. Appl. Polym. Sci..

[57] Ferrari AC (2007). Raman spectroscopy of graphene and graphite: Disorder, electron–phonon coupling, doping and nonadiabatic effects. Solid State Commun..

[58] Ferrari AC (2000). Raman spectrum of graphene and graphene layers. Phys. Rev. B.

[59] Ferrari AC, Robertson J (2001). Interpretation of infrared and Raman spectra of amorphous carbon nitrides. Phys. Rev. B.

[60] Thomsen C, Reich S (2003). Double resonant Raman scattering in graphite. Phys. Rev. Lett..

[61] Giovani P (2017). Few layer reduced graphene oxide: Evaluation of the best experimental conditions for easy production. Mater. Res..

[62] Sahoo SK, Archana M (2014). Fast and cost-effective electrochemical synthesis of few layer graphene nanosheets. NANO.

[63] Aladekomo JB, Bragg RH (1990). Structural transformations induced in graphite by grinding: Analysis of 002 X-ray diffraction line profiles. Carbon.

[64] Smidstrup S (2019). QuantumATK: An integrated platform of electronic and atomic-scale modelling tools. J. Phys. Condensed Matter.

[65] Perdew JP, Burke K, Ernzerhof M (1996). Generalized gradient approximation made simple. Phys. Rev. Lett..

[66] Tran F, Blaha P (2009). Accurate band gaps of semiconductors and insulators with a semilocal exchange-correlation potential. Phys. Rev. Lett..

[67] Liu DC, Nocedal J (1989). On the limited memory BFGS method for large scale optimization. Math. Program..

[68] Luo G (2013). Hole defects and nitrogen doping in graphene: Implication for supercapacitor applications. ACS Appl. Mater. Interfaces.

[69] Roy-Mayhew JD, Aksay IA (2014). Graphene materials and their use in dye-sensitized solar cells. Chem. Rev..

[70] Santhibhushan B, Soni M, Srivastava A (2017). Optical properties of boron-group (V) hexagonal nanowires: DFT investigation. Pramana.

[71] Chopra S, Maidich L (2014). Optical properties of pure graphene in various forms: A time dependent density functional theory study. RSC Adv..

[72] Surana K (2018). Studies on polarization effect of polyethylene-based polymer electrolyte in dye and quantum dot sensitized solar cells. Appl. Nanoscience.

[73] Yang N (2010). Two-dimensional graphene bridges enhanced photoinduced charge transport in dye-sensitized solar cells. ACS Nano.

[74] Zhan C (2015). Quantum effects on the capacitance of graphene-based electrodes. J. Phys. Chem. C.

[75] Ponomarenko LA (2010). Density of states and zero Landau level probed through capacitance of graphene. Phys. Rev. Lett..

[76] Zheng J, Suh S (2019). Strategies to reduce the global carbon footprint of plastics. Nat. Clim. Change.

[77] Singh PK (2011). Present status of solid state photoelectrochemical solar cells and dye sensitized solar cells using PEO-based polymer electrolytes. Adv. Nat. Sci. Nanosci. Nanotechnol..

[78] Surana K (2019). Utilizing reduced graphene oxide for achieving better efficient dye sensitized solar cells. J. Alloys Compounds.

